# Autism Spectrum Disorder and Disruptive Behavior Disorders Comorbidities Delineate Clinical Phenotypes in Attention-Deficit Hyperactivity Disorder: Novel Insights from the Assessment of Psychopathological and Neuropsychological Profiles

**DOI:** 10.3390/jcm9123839

**Published:** 2020-11-26

**Authors:** Gianluca Sesso, Chiara Cristofani, Stefano Berloffa, Paola Cristofani, Pamela Fantozzi, Emanuela Inguaggiato, Antonio Narzisi, Chiara Pfanner, Federica Ricci, Annalisa Tacchi, Elena Valente, Valentina Viglione, Annarita Milone, Gabriele Masi

**Affiliations:** 1 Department of Clinical and Experimental Medicine, University of Pisa, 56126 Pisa, Italy; gianse91@gmail.com; 2 IRCCS Stella Maris, Scientific Institute of Child Neurology and Psychiatry, Calambrone, 56128 Pisa, Italy; chiara.cristofani@fsm.unipi.it (C.C.); stefano.berloffa@fsm.unipi.it (S.B.); paola.cristofani@fsm.unipi.it (P.C.); pamela.fantozzi@fsm.unipi.it (P.F.); emanuela.inguaggiato@fsm.unipi.it (E.I.); antonio.narzisi@fsm.unipi.it (A.N.); chiara.pfanner@fsm.unipi.it (C.P.); federica.ricci@fsm.unipi.it (F.R.); annalisa.tacchi@fsm.unipi.it (A.T.); elena.valente@fsm.unipi.it (E.V.); valentina.viglione@fsm.unipi.it (V.V.); gabriele.masi@fsm.unipi.it (G.M.)

**Keywords:** attention-deficit hyperactivity disorder, autism spectrum disorder, oppositional defiant disorder, conduct disorder, neurodevelopment, disruptive behavior, executive functions, BRIEF, CBCL, children

## Abstract

Although childhood-onset psychiatric disorders are often considered as distinct and separate from each other, they frequently co-occur, with partial overlapping symptomatology. Autism spectrum disorder (ASD) and attention-deficit/hyperactivity disorder (ADHD) commonly co-occur with each other and with other mental disorders, particularly disruptive behavior disorders, oppositional defiant disorder/conduct disorder (ODD/CD). Whether these associated comorbidities represent a spectrum of distinct clinical phenotypes is matter of research. The aim of our study was to describe the clinical phenotypes of youths with ADHD with and without ASD and/or ODD/CD, based on neuropsychological and psychopathological variables. One-hundred fifty-one participants with ADHD were prospectively recruited and assigned to four clinical groups, and assessed by means of parent-reported questionnaires, the child behavior checklist and the behavior rating inventory of executive functions. The ADHD alone group presented a greater impairment in metacognitive executive functions, ADHD+ASD patients presented higher internalizing problems and deficits in Shifting tasks, and ADHD+ODD/CD subjects presented emotional-behavioral dysregulation. Moreover, ADHD+ASD+ODD/CD individuals exhibited greater internalizing and externalizing problems, and specific neuropsychological impairments in the domains of emotional regulation. Our study supports the need to implement the evaluation of the psychopathological and neuropsychological functioning profiles, and to characterize specific endophenotypes for a finely customized establishment of treatment strategies.

## 1. Introduction

Attention deficit and hyperactivity disorder (ADHD) is one of the most frequent reasons for consultation in the context of mental health services for minors, causing significant impairment in various life contexts from childhood to adolescence and adulthood [1,2,3]. Despite previous attempts to characterize its multifaceted nature, the considerable heterogeneity of its clinical presentations still remains a major subject of debate among clinicians [1]. One of the aspects of this heterogeneity is the comorbidity between ADHD and other psychiatric disorders in at least 60% of patients, mostly other neurodevelopmental conditions—particularly autism spectrum disorders (ASD), intellectual disabilities, Tourette syndrome, and motor coordination disorders [1,4,5,6,7,8]; and disruptive behavior disorders—such as oppositional defiant disorder (ODD) and conduct disorder (CD); but also internalizing disorders—especially anxiety and mood disorders. The coexistence of clinical comorbidities in the context of ADHD represents a serious matter especially in terms of early detection. Consequently, therapeutic interventions are even more challenging to define, with the risk of being less specific and less effective.

The impact of comorbidities in ADHD patients can be substantial. For instance, the severity of ADHD symptomatology is further worsened, in terms of emotional and behavioral problems, when ASD phenotype overlaps along with its adaptive impairment [9,10,11,12]. Particularly, these patients exhibit more severe externalizing behaviors, and greater impairments in verbal working memory [10]. A dual diagnosis of ADHD and ASD is now allowed according to the Diagnostic and Statistical Manual of Mental Disorders—Fifth Edition (DSM-5) criteria [13], with rates of co-occurrence of up to 42% [14,15]. On the other hand, ADHD children with comorbid ODD and/or CD are characterized by higher rates of learning difficulties and school problems, including neglect, expulsion, and dropouts from school [16,17], and lower performances in visual–motor integration and visuo-spatial tasks [18]. Additionally, they are more prone, in adulthood, to develop drug abuse, and to be engaged in criminal behaviors and antisocial conduct [19,20].

Recent studies have raised the question of whether ADHD with associated comorbidities is merely characterized by an increased symptoms variety with enhanced impairment severity, or it represents a spectrum of distinct phenotypes [21]. Interestingly, the identification of clinical phenotypes is a developing area of research aimed at best characterizing the multifaceted aspect of neurodevelopmental disorders. Current research is focused on the fine investigation of some dimensional constructs, such as executive function (EF) deficits, which are well known to characterize the neuropsychological profiles of affected individuals [21]. Indeed, studies comparing EF in ADHD with different comorbidities have identified specific executive profiles across different clinical phenotypes. It is still unclear, however, whether these neuropsychological difficulties are either trans-diagnostic or specific to that particular clinical entity.

EF deficits in children with ADHD and/or ASD feature some shared characteristics, as shown by a recent meta-analysis by Craig and colleagues [22], including 26 studies that used different assessment measures of EF. Although the results of such studies are not entirely consistent, the authors identified overlapping impairments in EF profiles—for instance, attention deficits significantly higher both in ADHD alone and ADHD+ASD patients. While performances in working memory and fluency tasks did not differ between the clinical groups, inhibition and cognitive flexibility appeared more impaired in ASD and ADHD+ASD children. Therefore, ADHD+ASD group may show a distinct and more severe dysexecutive profile, characterized by the presence of characteristic deficits of both disorders. Additionally, a more recent study by Berenguer and colleagues [23] assessed EF profiles in children with ADHD, ASD, and ADHD+ASD, comparing them with a control group, by means of the Behavior Rating Inventory of Executive Function (BRIEF) scale. Results revealed alterations in most BRIEF variables in all three clinical groups, with the exception of inhibit and material organization subscales, which appeared significantly affected only in the two groups with symptoms of ADHD. Moreover, ADHD alone and ADHD+ASD patients showed higher deficits in the working memory, plan/organize and monitor subscales than ASD group.

On the other hand, EF deficits have also been identified in children with ODD/CD, and they partially overlap with those typically found in ADHD patients [24,25]. Some results suggest that the executive dysfunction of children with ADHD and ODD/CD might be milder than that of children with ADHD alone [26]. Contradictory findings showed, however, that EF profiles of children with ADHD+ODD/CD were impaired, especially in terms of inhibition, more slightly [27] or more severely [28], than those of children with ADHD alone. Another study assessing EF by means of different environmentally valuable tools [29] proved that adolescents with ADHD+ODD/CD are less inclined to identify effective strategies and control behavioral responses, when compared to individuals with ODD/CD only. A study by Qian and colleagues [30] compared participants with ADHD and ADHD+ODD/CD to a healthy control group using the behavior rating inventory of executive function—second version (BRIEF-2) [31], showing that the comorbid group got worse scores in inhibit, shift, and emotional control subscales. Interestingly, Hobson et al. [32] confirmed overlapping profiles in metacognitive EF profile, assessed with standardized performance test, for individuals with ADHD and ODD/CD, who share similar difficulties in tolerating delayed rewards, but showed greater impairment in emotional regulation EF in ODD/CD group. Both the studies suggest that children with ADHD and ODD/CD may present a more pronounced impairment in emotional, rather than in cognitive control. From that perspective, a fairly simplistic but clinically useful distinction among two major dimensions of EF, namely, between “cold” and “hot” EF, has been proposed [33]. While the former are more strictly related to metacognitive skills such as working memory, programming, and organization attitudes, the latter refer to processes underlying the affective modulation of behavioral responses, or in other words, the emotional regulation domain.

Only a few studies have investigated EF profiles among young people with ADHD and comorbid ASD and/or ODD/CD. A recent study by Leno et al. [34] aimed at comparing different EF tasks’ performances in three clinical groups by controlling for conduct problems and ADHD symptoms. The authors concluded that only the ADHD+ASD group exhibited reduced inhibition in the go/no-go task, while all three clinical groups demonstrated greater reaction time variability than the control group; similarly, both ADHD+ODD/CD and ADHD+ASD groups showed an increase in impulse responses, whereas no differences could be detected in cognitive flexibility and switch. Finally, a study by Tye and colleagues [35], showed that stronger callous-unemotional traits in children with ASD improve performance in the go/no-go task, highlighting greater skills in inhibition processes.

Based on these findings, we hypothesize that ADHD alone and ADHD with associated comorbidities represent distinct phenotypes, with suitable clusters of psychopathological and neuropsychological features. These psychopathological and neuropsychological indices may be useful not only for differentiating clinical subtypes of patients, but also for identifying specific intervention pathways. To test this hypothesis, the main aim of our preliminary study is to describe the clinical phenotypes of minors with ADHD in its “pure” presentation and with psychiatric comorbidities (ASD and/or ODD/CD), with respect to psychopathological and neuropsychological variables, to highlight similarities and differences among the groups. This study represents one of few literature reports that evaluates clinical phenotypes of children with ADHD+ASD+ODD/CD comorbidity. Furthermore, to the best of our knowledge, this is the first study assessing EF in such a cohort through parental reports, by means of BRIEF-2 questionnaire designed to explore EF of youths in daily contexts, related to everyday life behaviors. Furthermore, the BRIEF-2 inventory finely captures the multidimensional structure of EF, and particularly the distinction between cold (metacognition) and hot (emotional regulation) dimensions.

Therefore, we assessed psychopathological profiles using a dimensional approach for identifying phenotypic domains, representing meaningful variations across multiple domains of behaviors, with two broader dimensions, internalizing and externalizing behaviors [36,37,38].

## 2. Experimental Section

### 2.1. Participants and Diagnostic Procedures

Our study prospectively included 151 drug-naïve participants (137 boys, age range 6–18 years old, mean age 9.51 ± 2.64 years) recruited from March 2019 to December 2019 in the Department of Child and Adolescent Psychiatry of our third level hospital with a nation-wide catchment. The investigation was carried out in accordance with the latest version of the Declaration of Helsinki.

The diagnoses, based on DSM-5 diagnostic criteria, were made using historical information, and a structured clinical interview, the Italian version of the Schedule for Affective Disorders and Schizophrenia for School-Age Children—Present and Lifetime Version (K-SADS-PL) [39], administered by child psychiatry trainees under the supervision of the senior child psychiatrist. Diagnoses were finally confirmed by consensus of a multidisciplinary board.

The Italian version of the Wechsler Intelligence Scale for Children—Fourth Edition (WISC-IV) [40] was also routinely administered to all patients to assess the intellectual functioning. Moreover, the Italian versions of the Autism Diagnostic Observation Schedule—Second Edition (ADOS-2) [41] and of the Autism Diagnostic Interview—revised (ADI-R) [42] were administered only in those patients for whom an initial diagnosis of ASD according to DSM-5 criteria was discussed. Thus, they were used to assist clinicians in the diagnostic procedure and to either confirm or to question the ASD diagnoses by final consensus of our multidisciplinary team, as routinely performed in the Department according to international guidelines. All assessments were conducted by highly experienced psychologists specialized in child neuropsychological evaluations. 

### 2.2. Inclusion and Exclusion Criteria

Patients were included whether they received a diagnosis of ADHD with or without comorbid ASD, ODD/CD, or both. Other associated psychiatric conditions, such as anxiety and mood disorders and learning disabilities, were accepted but not primarily considered for analytical purposes. Exclusion criteria were as follows: a total WISC-IV IQ score lower than 70 points; younger than 6 years old or older than 18 years old; use of psychoactive medications; neurologic impairments or neurodegenerative conditions. All recruited patients met inclusion criteria and agreed to participate in the study after informed consent was obtained by parents. 

### 2.3. Clinical Groups

We identified four clinical groups in our sample: ADHD alone group, including 64 participants (12.5% girls, mean age 10.02 ± 2.49 years); comorbid ADHD+ASD group (hereinafter referred to as ADHD+ASD group), including 19 participants (5.26% girls, mean age 9.58 ± 2.69 years) who did not fulfill ODD/CD diagnostic criteria; comorbid ADHD+ODD/CD group (hereinafter referred to as ADHD+ODD/CD group), including 43 participants (9.3% girls, mean age 9.37 ± 2.95 years) who did not fulfill ASD diagnostic criteria; comorbid ADHD+ASD+ODD/CD group (hereinafter referred to as ADHD+ASD+ODD/CD group), including 25 participants (4% girls, mean age 8.40 ± 2.24 years) who fulfilled diagnostic criteria for both ODD/CD and ASD. These groups could present other associated psychiatric conditions, i.e., anxiety and mood disorders or learning disabilities. 

### 2.4. Measures

All patients were assessed with the Italian version of the child behavior checklist for ages 6 to 18 years (CBCL-6/18) [43], a 118-item scale, completed by parents, with 8 different syndromes scales, a total problem score, and two broad-band scores designated as internalizing problems and externalizing problems. The reliability coefficients (Cronbach’s alpha) were 0.82, 0.81, and 0.82, respectively [43]. 

The parents of all patients were asked to fill in the Italian version of the BRIEF-2 inventory [31]. The instrument is the updated version of the BRIEF questionnaire and provides a structured assessment of EF behaviors in everyday life environments, allowing the identification of helpful clinical manifestations in different contexts, i.e., home and school. Designed for ages 5 to 18, BRIEF-2 is available in three versions (parent-report, teacher-report, and self-report). In this study, the parent-reported version was used, which includes 63 items assessing the frequency of occurrence of common behaviors in everyday life settings. BRIEF-2 is a multidimensional measurement that investigates nine factors related to specific EFs: inhibit, self-monitor, shift, emotional control, initiate, working memory, plan/organize, task monitor, organization of materials. Three composite scales are also identifiable: behavioral regulation index (BRI) (including inhibit and self-monitor), emotional regulation index (ERI) (shift and emotional control), and cognitive regulation index (CRI) (initiate, working memory, plan/organize, task monitor, organization of materials). A global executive composite (GEC) score was also computed as the sum of the three composite indexes. 

### 2.5. Statistical Analysis

Statistical analyses were performed by means of MatLab^®^ (MathWorks, Natick, MA, USA) and RStudio^®^ (RStudio Inc., Boston, MA, USA) software. For each clinical variable with a continuous distribution, outliers were defined as observations lying outside the range between (first quartile − 1.5 * interquartile range) and (third quartile + 1.5 * interquartile range) and removed. For each BRIEF-2 subscale-related variable, observations were removed if the corresponding values for either the infrequency or the inconsistency scale were higher than the 99th percentile of normalized data. 

The χ^2^ test was used to detect significant differences (*p*-value < 0.05) for clinical and demographic categorial variables, such as gender and clinical comorbidities. When more than 20% of observations had expected frequencies less than 5, Fisher’s exact test was performed. Analyses of variance (ANOVA) was conducted to assess significant differences (*p*-value < 0.05) between clinical and demographic variables with continuous distribution. Homogeneity of variances across groups was checked using Levene’s test before applying ANOVA; all variables showed equal variances in the different groups (Levene’s test: *p*-value > 0.05), so that variance homogeneity assumption was reliably satisfied. A Tukey post-hoc test was utilized whenever the ANOVA led to a statistically significant result in order to retrieve significant comparisons between variables. 

Finally, we performed a principal component analysis (PCA) with Promax oblique rotation to empirically derive variables that were subsequently compared between clinical groups. All BRIEF-2 and CBCL-6/18 subscale variables were used to conduct the PCA—specifically, the inhibit, self-monitor, emotional control, shift, initiate, working memory, plan/organize, task-monitor, and organization of materials scales of former; and the anxious/depressed, withdrawn/depressed, social problems, somatic complaints and thought problems, attention problems, rule-breaking behavior, and aggressive behavior symptoms scales of the latter. A scree-plot was used to determine the appropriate number of components. Thus, a number (greater than 1) of components was extracted, and component scores for each participant were computed using regression approach. An ANOVA with Tukey’s post-hoc test was then conducted to assess significant differences between groups in the continuous data of the three components identified through the PCA. 

## 3. Results

### 3.1. Clinical and Demographic Characteristics 

As shown in Table 1, the four clinical groups did not differ in terms of age or gender. No significant difference emerged in WISC-IV scores, nor in full-scale intelligence quotient, nor in its four composite scores; nonetheless, all four groups scored appreciably lower in working memory and processing speed composites than the verbal comprehension and perceptual reasoning composites. Clinical comorbidities are also reported; as expected, mood disorders were more expressed in the ADHD+ASD+ODD/CD group than the ADHD group only, while anxiety disorders were more prevalent in the ADHD+ASD group than the ADHD only group. 

### 3.2. Child Behavior Checklist

**Figure 1:**
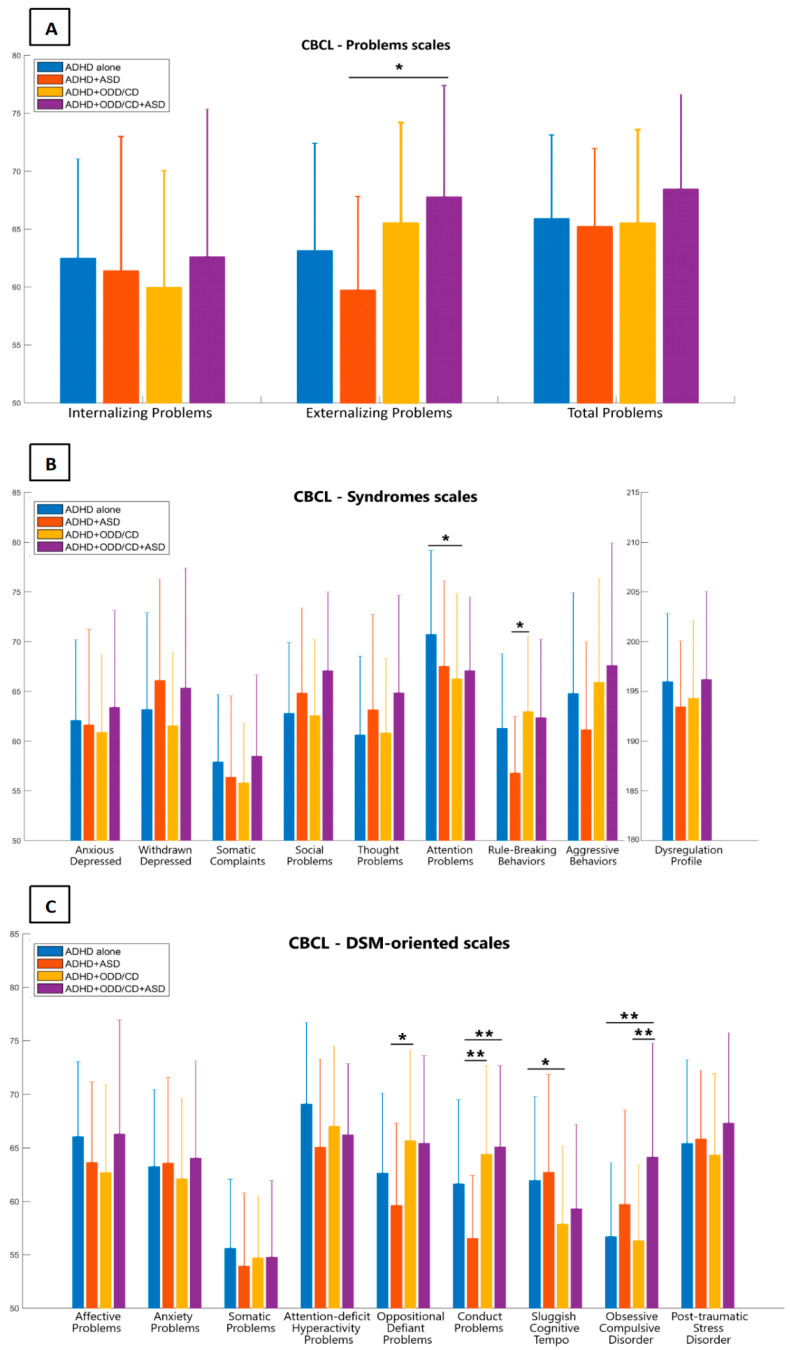
Scores obtained by the four clinical groups in the child behavior checklist questionnaire are illustrated: (**A**) problems scales, (**B**) syndromes scales, and (**C**) DSM-oriented scales are compared between ADHD alone group (blue bars), ADHD+ASD group (red bars), ADHD+ODD/CD group (yellow bars), and ADHD+ASD+ODD/CD group (purple bars). Graphs represent means with standard deviation bars. * *p*-values < 0.05, ** *p*-values < 0.01.

Only one outlier was removed from the analysis in the somatic complaints subscale of the CBCL questionnaire, belonging to the ADHD+ASD+ODD/CD group. No outliers were identified and removed based on interquartile range for all the other CBCL variables. Scores obtained by the four clinical groups and ANOVA statistics in the CBCL are illustrated in Figure 1A–C and Table 2. Significant differences were detected in the externalizing problems scale (Figure 1A) between ADHD+ASD (59.72 ± 8.10) and ADHD+ASD+ODD/CD (67.77 ± 9.62) groups, but no significant differences emerged for the internalizing and the total problems scales, though the mean scores of all four groups exceeded the clinical cut-offs for both. 

As for the syndromes scale (Figure 1B), a significant difference was found in the attention problems scale between ADHD alone (70.73 ± 8.44) and ADHD+ODD/CD groups (66.25 ± 8.55); in the rule-breaking behaviors scale the ADHD+ODD/CD group (62.97 ± 7.57) scored significantly higher than the ADHD+ASD group (56.78 ± 5.68). 

As for the DSM-oriented scales (Figure 1C), the ADHD+ODD/CD group scored significantly higher (65.67 ± 8.48) than the ADHD+ASD group (59.63 ± 7.68) in the oppositional-defiant problems scale, and significantly higher (64.39 ± 8.29) than the ADHD+ASD group (56.52 ± 5.89) in the conduct problems scale, as did the ADHD+ASD+ODD/CD group (65.09 ± 7.52). 

Finally, significant differences emerged in the sluggish cognitive tempo scale between ADHD alone (62.72 ± 9.13) and ADHD+ODD/CD groups (57.88 ± 7.26), and in the obsessive-compulsive problems scale between ADHD+ASD+ODD/CD (64.13 ± 10.63), and both ADHD alone (56.69 ± 6.91) and ADHD+ODD/CD groups (56.33 ± 7.02).

### 3.3. Behavior Rating Inventory of Executive Function

**Figure 2:**
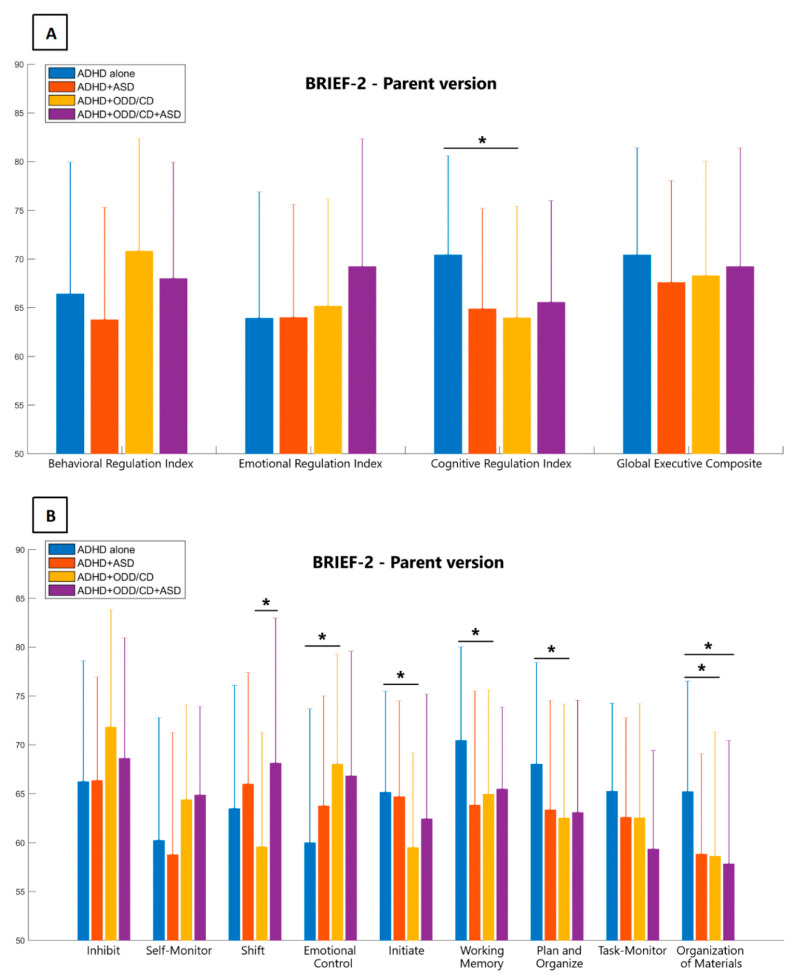
Scores obtained by the four clinical groups in the brief rating inventory of executive functions questionnaire are illustrated: (**A**) global indexes and (**B**) specific subscales are compared between ADHD alone group (blue bars), ADHD+ASD group (red bars), ADHD+ODD/CD group (yellow bars), and ADHD+ASD+ODD/CD group (purple bars). Graphs represent means with standard deviation bars. * *p*-values < 0.05.

No outliers were identified and removed based on BRIEF-2 variables’ interquartile ranges. Profiles of the scores obtained by the four clinical groups in the BRIEF-2 scales and ANOVA statistics are shown in Figure 2A,B and Table 3. As for the BRIEF-2 general indexes (Figure 2A), the only significant difference was found for the CRI, with higher scores obtained by ADHD alone group (70.41 ± 10.16) compared to ADHD+ODD/CD group (63.97 ± 11.41). It may be noticed that, although differences did not reach statistical significance, ODD/CD and ADHD+ASD+ODD/CD groups scored the highest in both BRI and ERI, exceeding cut-off values. Similarly, although differences among groups were not significant, all four groups presented elevated scores, exceeding the cut-off values, in the GEC scale.

As for the BRIEF-2 subscales (Figure 2B), inhibition, self-monitor, and task monitor subscales did not differentiate the four groups. The ADHD alone group scored significantly higher than the other three groups in working memory, plan/organize, and organization of materials subscales. The initiate subscale score was significantly higher in the ADHD alone group, compared to the ADHD+ODD/CD group, but not compared to the other two groups. The ADHD+ODD/CD group scored significantly higher than the ADHD alone in the emotional control scale, while the ADHD+ASD+ODD/CD group only approached statistical significance.

### 3.4. Principal Component Analysis

The PCA we performed led to a three-component solution explaining 59% of variance. As shown in Table 4, the first rotated component (RC1) included the cognitive regulation-related subscales (initiate, working memory, plan/organize, task-monitor, and organization of Materials) of the BRIEF-2 and the attention problems of the CBCL. The second rotated component (RC2) included the shift subscale of the BRIEF-2 and the internalizing problems-related subscales of the CBCL (anxious/depressed, withdrawn/depressed, social problems, somatic complaints, and thought problems). The third rotated component (RC3) included the behavioral regulation-related subscales (inhibit and self-monitor) and the emotional control subscale of the BRIEF-2, and the externalizing problems-related subscales of the CBCL (rule-breaking behavior and aggressive behavior). High scores represent greater impairment impairments in all variables included in the different components.

**Figure 3:**
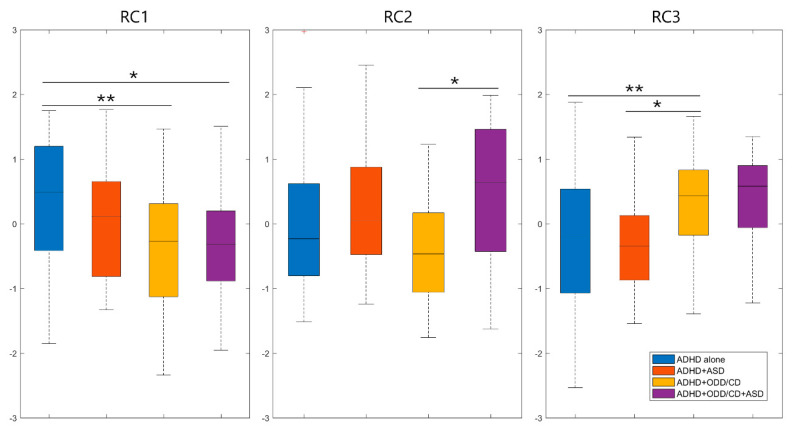
Scores obtained by the four clinical groups in the three components of the principal component analysis, as described in detail in the main text, are illustrated here: (**left**) RC1, (**middle**) RC2, and (**right**) RC3 are compared between ADHD alone group (blue bars), ADHD+ASD group (red bars), ADHD+ODD/CD group (yellow bars), and ADHD+ASD+ODD/CD group (purple bars). Boxplots represent medians and first and third quartiles with minimum/maximum bars. * *p*-values < 0.05, ** *p*-values < 0.01. Abbreviations: RC1 = first rotated component; RC2 = second rotated component; RC3 = third rotated component.

As illustrated in Figure 3, the ADHD alone group scored the highest in the RC1 component and significantly differed from the ADHD+ODD/CD and the ADHD+ASD+ODD/CD groups. As for the RC2 component, a significant difference emerged between the ADHD+ASD+ODD/CD and the ADHD+ODD/CD groups, the former scoring higher than the latter. On the contrary, the ADHD+ODD/CD group scored the highest in the RC3 component, significantly differing from the ADHD alone and the ADHD+ASD groups, while the difference approached statistical significance between the ADHD+ASD+ODD/CD and the ADHD groups. 

## 4. Discussion

The main aim of the present study was to identify possible phenotypes of patients with ADHD, ASD, and ODD/CD, based on EF and psychopathological domains, in order to better characterize their diagnostic frameworks. To the best of our knowledge, this is the first study assessing EF through parental reports, by means of the BRIEF-2 questionnaire, in a clinical sample of youth with comorbid conditions.

The BRIEF-2 questionnaire provides a structured assessment of EF behaviors in everyday life environments, by means of a multi-comprehensive assessment of different aspects of child behavior. A large number of world-wide clinical trials and case-control studies are indeed available to derive specific normative data based on age and gender. For these reasons, we chose the BRIEF-2 questionnaire as a valuable instrument for the assessment of EF in our sample.

The four groups in our sample were similar in terms of age, gender, and cognitive abilities. Our analyses demonstrated in the ADHD alone group a specific pattern of impairment in attentive and metacognitive domains, as revealed by CBCL questionnaire and BRIEF-2 inventory (cognitive regulation, working memory, plan/organize, organization of materials, initiate), with greater impairment in the domains of the so-called “cold” EF. These features appear more evident in the ADHD alone group when compared to those associated with comorbid conditions, which seems to support the executive dysfunction theory of ADHD [25,44,45]. Other features (i.e., inhibition, self-monitoring, shift, and task monitoring) do not seem to be affected by comorbidities, as they are indifferently present both in ADHD alone and when it is associated with ODD/CD and/or ASD.

As expected, when ADHD is comorbid with ODD/CD, it is associated not only with attention problems (but not with the sluggish cognitive tempo subscale), but also with rule breaking behavior [16]. Externalizing problems score is not significantly higher in ADHD+ODD/CD compared to ADHD alone and ADHD+ASD groups, and only the association ADHD+ASD+ODD/CD presents a significantly higher externalizing problems score. In contrast, the ADHD+ASD condition is prevalently associated with higher obsessive-compulsive traits and sluggish cognitive tempo, lower scores in rule breaking behaviors and conduct problems, and when associated with ODD/CD, lower shift abilities, compared to ODD/CD without ASD.

The PCA supports these findings. Indeed, the ADHD alone group is associated with the attention problems of CBCL, and with the cognitive regulation-related subscales (initiate, working memory, plan/organize, task-monitor, and organization of materials) of BRIEF-2. The triple comorbidity ADHD+ASD+ODD/CD is associated with both RC2 and RC3. However, the RC2 (internalizing problems-related subscales of CBCL, anxious/depressed, withdrawn/depressed, social problems, somatic complaints, and thought problems, and the shift subscale of BRIEF-2) is prevalently associated with both ADHD+ASD and ADHD+ASD+ODD/CD, with a prevalent contribution of the ASD component. On the other hand, the RC3 (externalizing problems-related subscales of the CBCL; rule-breaking behavior and aggressive behavior, inhibit, and self-monitor; and the emotional control subscale of the BRIEF-2) is prevalently associated with the ADHD+ASD+ODD/CD and ADHD+ODD/CD, with a prevalent contribution of the ODD/CD component.

While the profile of the ADHD+ODD/CD is consistent with previous findings regarding the increased severity of both externalizing symptoms and emotional dysregulation [46,47,48], the profile of ADHD participants with comorbid ASD is more difficult to interpret, and consistent with the notion that the ADHD+ASD phenotype does not simply reflect the sum of the symptoms of the two disorders [9,49]. Our results suggest that the presence of ASD traits modulates the severity of the externalizing symptoms, further increasing aggressive and shattering behaviors. Indeed, externalizing symptoms are lowest when ASD is associated with ADHD, even compared to ADHD alone, and are highest when ASD is associated with both ADHD and ODD/CD, thereby worsening the externalizing impact of this association. However, in line with previous studies [50], the principal component analysis we performed suggests that the ADHD+ASD+ODD/CD association further increases internalizing problems (anxiety, mood symptoms, and social problems). Consistently, Cooper at al. (2014) correlated the presence of autistic traits in children with ADHD with a greater severity of behavioral symptoms and anxiety symptoms.

In summary, our study highlighted that, compared to ADHD alone, comorbid ODD/CD and ASD worsen externalizing and internalizing symptoms’ severity, and the neuropsychological profile, but with different patterns. ADHD alone appears to be characterized by a more specific impairment of metacognitive (“cold”) functioning and attentive capacities. On the other hand, comorbid ODD/CD seems more significantly related to “hot” executive dysfunctions and emotional self-regulation. Less consistent findings have been found regarding the implications of ASD comorbidity, namely, a more selective impairment in shifting, possibly related to their low cognitive flexibility.

Our data are in line with novel approaches to the classical categorical nosographic entities, using dimensional psychopathological and neuropsychological tools, in a transdiagnostic fashion, possibly with supporting evidence from biological markers, to understand the common patterns of neural dysfunction that link apparently different disorders, including ADHD and ASD [51]. Within this framework, defining a new approach to understanding mental illness, the Research Domain Criteria (RDoC) project [52] presented mixed dimensional abnormalities based on brain circuits and connectivity, which are conceptualized as underlying dysfunctions that can contribute to clinically diverging mental disorders, such as ADHD and ASD, as recently shown [53,54]. Our study does not provide biological markers, and dimensional and psychopathological tools were applied to behavioral syndromes according to the “old” DSM-5 categories. This “false assumption” [55] has been limited so far to the extension of this novel approach to child and adolescent psychiatry [56]. However, the intent of RDoC is not to become a new diagnostic classification system, but it may be useful for refining current diagnostic classifications through the lens of fundamental components, i.e., executive functions, that cut across diagnoses [56]. This approach may be also functional for understand the heterogeneity within a single DSM disorder, based on neuropsychological, psychopathological, or temperamental dimensions.

Our study shows, however, a number of limitations. Importantly, a marked discrepancy in the sizes of the four groups, particularly between the ADHD alone group and those associated with comorbid ASD. Furthermore, these groups were not recruited through an age-matching protocol design, though no significant difference in age emerged when comparing them by means of statistical tests. Additionally, we did not include a control group of healthy children. Therefore, future studies with larger age- and sex-matched samples and healthy controls could be carried out to support our results. 

Moreover, an ecological parent report-based measure of EF was used in the present study. It should be noted that low correlations have been reported between performance measures and behavioral rating scales of EF in ADHD and ASD research; however, task-based measures capture only limited facets of the EF system in a limited temporal span, while neglecting the integrated multidimensional decisional process related to and based on a priority-based strategic analysis performed by the individual, which is what daily life situations often require. From this perspective, ecological measures are important to foresee the severity of dysfunction in daily life situations experienced by children at home and school, which are important settings wherein parents and teachers assessing the essential expressions of executive functioning may provide a valuable amount of information which could help clinicians in their measurements.

Another limitation of our study is that it did not include the “limited prosocial emotion” specifier. Further research could be performed, including using more participants showing high levels of callous-unemotional traits, in order to possibly identify further phenotypes of ADHD with comorbid disruptive behavior disorders. Similarly, we could not ascertain the presence of subthreshold symptoms within the different clinical domains explored by the K-SADS interview and the ADOS-2 observation; subthreshold symptoms could be usefully considered in a multidimensional perspective in future studies. Unfortunately, we could not provide teacher-rated measures of ADHD symptoms to compare parent reports, though educational observations were performed for all patients. Finally, we did not correct our tests for multiple comparisons, as all comparisons we performed would not survive such correction with a statistical threshold below 0.001. Nonetheless, given the exploratory nature of our study and the relatively limited sample size, we believed it more convenient to keep all statistically significant comparisons while waiting for future larger studies aimed at corroborating—or invalidating—our results.

## 5. Conclusions

Our study provides a further contribution to a better understanding of the clinical manifestations of ADHD and comorbidities with ASD and/or ODD/CD, and suggests that specific comorbidities might help in the selection of the treatment strategies. Our findings support the need to associate, within the clinical assessment and diagnostic framework, the evaluation of psychiatric comorbidities and neuropsychological functioning, with more thoroughly characterized specific clinical phenotypes, i.e., by means of standardized neuropsychological tests in clinical settings.

## Figures and Tables

**Table 1:** Demographic and clinical data.

	ADHD	ADHD+ASD	ADHD+ODD/CD	ADHD+ASD+ODD/CD	*p*-Values
Subjects ^a^	64 (42.38)	19 (12.58)	43 (28.48)	25 (16.56)	
Age ^b^	10.02 ± 2.49	9.58 ± 2.69	9.37 ± 2.95	8.40 ± 2.24	0.0760
*Adolescents* ^a^	19 (29.69)	6 (31.58)	10 (23.26)	4 (16)	0.5238
Gender ^a^	8 (12.5)	1 (5.26)	4 (9.3)	1 (4)	0.6998
WISC-IV					
*VCI* ^b^	104.89 ± 17.41	104.76 ± 17	104.5 ± 14.61	107.32 ± 19.24	0.9440
*PRI* ^b^	99.11 ± 12.76	104.76 ± 17.07	103.82 ± 18.17	107.77 ± 16.19	0.2860
*WMI* ^b^	87.07 ± 13.38	88.07 ± 7.32	88.95 ± 11.53	90.33 ± 17.45	0.8710
*PSI* ^b^	83.56 ± 15.28	83.73 ± 13.97	89.47 ± 18.46	83.1 ± 14.89	0.5470
*FSIQ* or *GAI* ^b^	93 ± 14.98	92.69 ± 17	96.86 ± 16.05	98.94 ± 18.06	0.5440
Comorbidities					
*Mood Dis* ^a^	8 (12.50) *	7 (36.84)	14 (32.56)	13 (52.00) *	0.0011 *
*Anxiety Dis* ^a^	7 (10.94) *	8 (42.11) *	7 (16.28)	8 (32.00)	0.0087 *

Data are presented either as (a) number (percentage) for dichotomous variables or (b) mean ± standard deviation for continuous variables. * *p*-values < 0.05. Abbreviations: VCI = verbal comprehension index; PRI = perceptual reasoning index; WMI = working memory index; PSI = processing speed index; FSIQ = full-scale intelligence quotient; GAI = general ability index; Dis = disorders.

**Table 2:** CBCL-6/18 scales.

*CBCL – 6/18*	ADHD	ADHD+ASD	ADHD+ODD/CD	ADHD+ASD+ODD/CD	F Value	*p*-Values
Internalizing P	62.47 ± 8.59	61.37 ± 11.62	59.95 ± 10.09	62.61 ± 12.75	0.612	0.608
Externalizing P	63.13 ± 9.28	59.72 ± 8.10	65.51 ± 8.69	67.77 ± 9.63	3.222	0.025 *
Total Problems	65.89 ± 7.24	65.22 ± 6.73	65.49 ± 8.09	68.45 ± 8.18	0.896	0.445
Anxious/Dep	62.09 ± 8.09	61.63 ± 9.62	60.88 ± 7.84	63.41 ± 9.72	0.455	0.714
Withdrawn/Dep	63.19 ± 9.73	66.11 ± 10.19	61.56 ± 7.37	65.35 ± 12.07	1.361	0.257
Somatic C	57.90 ± 6.81	56.37 ± 8.17	55.81 ± 5.99	58.48 ± 8.16	1.121	0.343
Social Problems	62.78 ± 7.14	64.84 ± 8.53	62.59 ± 7.66	67.09 ± 7.88	2.239	0.086
Thought P	60.63 ± 7.91	63.16 ± 9.59	60.84 ± 7.47	64.87 ± 9.79	1.814	0.147
Attention P	70.73 ± 8.45	67.53 ± 8.59	66.26 ± 8.55	67.10 ± 7.39	2.786	0.043 *
Rule-Breaking B	61.28 ± 7.51	56.79 ± 5.68	62.98 ± 7.57	62.36 ± 7.91	3.252	0.024 *
Aggressive B	64.78 ± 10.16	61.16 ± 8.85	65.93 ± 10.46	67.61 ± 12.35	1.448	0.231
DP Index	197.94 ± 20.62	190.32 ± 19.90	192.93 ± 23.40	198.64 ± 26.56	0.930	0.428
Affective P	66.05 ± 7.02	63.63 ± 7.54	62.70 ± 8.18	66.30 ± 10.62	1.885	0.135
Anxious P	63.25 ± 7.20	63.58 ± 7.99	62.12 ± 7.54	64.04 ± 9.09	0.380	0.767
Somatic P	55.60 ± 6.47	53.95 ± 6.83	54.72 ± 5.79	54.78 ± 7.15	0.389	0.761
ADHD P	69.09 ± 7.62	65.06 ± 8.24	67.02 ± 7.48	66.22 ± 6.65	1.870	0.137
ODD P	62.63 ± 7.47	59.63 ± 7.68	65.67 ± 8.48	65.43 ± 8.17	3.316	0.022 *
Conduct P	61.63 ± 7.86	56.53 ± 5.89	64.40 ± 8.29	65.09 ± 7.58	5.693	0.001 **
SCT	61.94 ± 7.86	62.72 ± 9.13	57.88 ± 7.27	59.30 ± 7.88	2.922	0.036 *
OCD P	56.69 ± 6.91	59.71 ± 8.82	56.33 ± 7.02	64.14 ± 10.63	5.952	0.001 **
PTSD P	65.40 ± 7.85	65.83 ± 6.44	64.33 ± 7.56	67.32 ± 8.44	0.741	0.529

Data are presented as mean ± standard deviation. * *p*-values < 0.05; ** *p*-values < 0.01. Abbreviations: B = behaviors; C = complaints; Dep = depressed; DP = dysregulation profile; OCD = obsessive compulsive disorder; P = problems; PTSD = post-traumatic stress disorder; SCT = sluggish cognitive tempo.

**Table 3:** BRIEF-2 scales.

*BRIEF-2*	ADHD	ADHD+ASD	ADHD+ODD/CD	ADHD+ASD+ODD/CD	F Value	*p*-Values
ERI	63.92 ± 12.95	64.00 ± 11.60	65.16 ± 11.04	69.22 ± 13.10	1.086	0.358
BRI	66.40 ± 13.51	63.76 ± 11.54	70.82 ± 11.53	68.00 ± 11.93	1.562	0.201
CRI	70.42 ± 10.16	64.88 ± 10.30	63.97 ± 11.41	65.57 ± 10.42	3.551	0.016 *
GEC	70.40 ± 11.00	67.59 ± 10.47	68.29 ± 11.73	69.22 ± 12.17	0.426	0.735
Inhibition	66.23 ± 12.37	66.35 ± 10.58	71.82 ± 12.03	68.61 ± 12.34	1.809	0.149
Self-Monitor	60.22 ± 12.55	58.76 ± 12.47	64.39 ± 9.68	64.87 ± 9.06	2.036	0.112
Shift	63.48 ± 12.58	66.00 ± 11.37	59.58 ± 11.69	68.13 ± 14.85	2.408	0.059 ^§^
Emotional C	59.98 ± 13.69	63.76 ± 11.22	68.03 ± 11.25	66.83 ± 12.75	3.698	0.014 *
Initiate	65.15 ± 10.34	64.71 ± 9.80	59.50 ± 9.66	62.43 ± 12.73	2.423	0.059 ^§^
Working M	70.45 ± 9.57	63.82 ± 11.67	64.95 ± 10.70	65.48 ± 8.37	3.705	0.013 *
Plan/Organize	68.02 ± 10.40	63.35 ± 11.17	62.5 ± 11.65	63.09 ± 11.48	2.549	0.050 *
Org of Materials	65.21 ± 11.31	58.82 ± 10.25	58.61 ± 12.70	57.83 ± 12.61	3.802	0.012 *
Task Monitor	65.24 ± 9.01	62.59 ± 10.18	62.52 ± 11.68	59.35 ± 10.10	2.032	0.112

Data are presented as mean ± standard deviation. ^§^ *p*-values < 0.06; * *p*-values < 0.05. Abbreviations: C = control; M = memory; Org = organization.

**Table 4:** Principal component analysis.

	RC1	RC2	RC3
BRIEF-2—Plan/Organize	**0.8420**	0.2389	0.1921
BRIEF-2—Task Monitor	**0.8019**	0.0576	0.2487
BRIEF-2—Working Memory	**0.7848**	0.0050	0.1382
BRIEF-2—Organization of Materials	**0.6903**	−0.0165	0.1760
BRIEF-2—Initiate	**0.6900**	0.3306	0.0691
CBCL—Attention Problems	**0.5421**	0.3778	0.2531
CBCL—Anxious/Depressed	0.0162	**0.8254**	0.1641
CBCL—Somatic Complaints	0.2113	**0.7475**	−0.0119
CBCL—Social Problems	0.0169	**0.7259**	0.3128
CBCL—Withdrawn/Depressed	0.1771	**0.7107**	0.1029
CBCL—Thought Problems	0.0625	**0.6922**	0.2486
BRIEF-2—Shift	0.4150	**0.5162**	0.1894
BRIEF-2—Inhibit	0.2657	0.0520	**0.8497**
CBCL—Rule-Breaking Behaviors	0.2006	0.1169	**0.7782**
CBCL—Aggressive Behaviors	0.1491	0.3767	**0.7615**
BRIEF-2—Emotional Control	0.1613	0.3199	**0.6968**
BRIEF-2—Self-Monitor	0.1731	0.1165	**0.6913**
Unadjusted Eigenvalue	6.6114	2.2253	1.7460
Adjusted Eigenvalue	5.9195	1.6879	1.3212
Proportion Variance	0.2151	0.2121	0.1954
Cumulative Variance	0.2151	0.4271	0.6225

Data are presented as z-scores. Abbreviations: RC1 = first rotated component; RC2 = second rotated component; RC3 = third rotated component. Component loadings > 0.5 are shown in bold.
